# Supportive psychological intervention on psychological disorders in clinical medicine students with English Learning Difficulties: A protocol of systematic review

**DOI:** 10.1097/MD.0000000000023196

**Published:** 2020-11-20

**Authors:** Hong-li Wen, Chen Feng, Shu-ling Zhang, Xiao-wei Li

**Affiliations:** a Department of English, Mudanjiang Medical University; b First Ward of Orthopedics Department, The Affiliated Hongqi Hospital of Mudanjiang Medical University, Mudanjiang, China.

**Keywords:** psychological disorder, case-controlled study, effect

## Abstract

**Background:**

This study aims to examine the effect of supportive psychological intervention (SPI) on psychological disorders (PD) in clinical medicine students (CMS) with English Learning Difficulties (ELD).

**Methods:**

We will perform a comprehensive literature search from the following databases: Cochrane Library, MEDLINE, EMBASE, Allied and Complementary Medicine Database, Chinese Biomedical Literature Database, and China National Knowledge Infrastructure. All databases will be performed from their inception to the present without language limitation by 2 independent reviewers. We will also look for grey literature, such as conference proceedings, dissertations or theses. Newcastle-Ottawa Scale will be used to assess study quality, and RevMan 5.3 software will be applied to carry out statistical analysis.

**Results:**

This study will summarize the most recent evidence to assess the effect of SPI on PD in CMS with ELD.

**Conclusion:**

This study may provide helpful evidence of SPI on PD in CMS with ELD.

**OSF registration number:**

osf.io/tah2s.

## 1 Introduction

Medical education is often considered as very stressful.^[1–4]^ It often causes declines in subjective well-being in clinical medical students (CMS),^[5,6]^ because of the heavy workload, fear of failing exams for those students, especially for them with English Learning Difficulties (ELD).^[7–10]^ Research indicates that CMS with ELD often accompany serious psychological disorders (PD), including depression, anxiety, stress, and pressure.^[11–16]^ Fortunately, studies suggested that supportive psychological intervention (SPI) can help relief PD in CMS with ELD.^[1,7,13,17–20]^ However, there still no study specifically investigate the effect of SPI on PD in CMS with ELD. Thus, this study firstly examines the effect of SPI on PD in CMS with ELD.

## 2 Methods

### 2.1 Study registration

We have registered this study on OSF (osf.io/tah2s); and we have reported it according to the Preferred Reporting Items for Systematic Reviews and Meta-Analysis Protocol statement guidelines.

### 2.2 Eligibility criteria

#### 2.2.1 Types of studies

In this study, we will only include case-controlled study (CCS) that explores the effect of SPI on PD in CMS with ELD. We will exclude animal studies, case studies, reviews, and studies without controls.

#### 2.2.2 Types of interventions

In the intervention group, all participants utilized SPI as their management.

In the control group, all patients used any therapy, but not any forms of SPI.

#### 2.2.3 Types of participants

We will include participants with ELD who were diagnosed as PD (including depression, anxiety, and pressure), regardless race, gender, and economic status.

#### 2.2.4 Types of outcomes

Primary outcomes comprise of depression (as measured by any scale, such as Self-Rating Depression Scale) and anxiety (as assessed by any tool, such as Self-Rating Anxiety Scale).

Secondary outcomes include stress, pressure, quality of life, and any unexpected adverse events.

### 2.3 Information sources and search procedure

We will perform a comprehensive literature search from the inception to the present in Cochrane Library, MEDLINE, EMBASE, Allied and Complementary Medicine Database, Chinese Biomedical Literature Database, and China National Knowledge Infrastructure. All searches will be conducted without language restriction. The search strategy will be applied for MEDLINE and is presented in Table 1. We will adapt similar search strategy to other electronic databases. In addition, we will also check grey literature, including conference proceedings, dissertations or theses, and reference lists of included studies.

**Table 1:** Search strategy of MEDLINE.

Number	Search terms
1	Medical students
2	College students
3	University students
4	Young adults
5	English learning difficulties
6	Language learning difficulties
7	Academic learning difficulties
8	Or 1–7
9	Psychological disorder
10	Psychological condition
11	Psychological problem
12	Depression
13	Anxiety
14	Stress
15	Pressure
16	Or 9–15
17	Supportive psychological intervention
18	Supportive counseling
19	Treatment
20	Management
21	Therapy
22	Or 17–21
23	Case-control studies
24	Case-referent study
25	Case-control
26	Case-comparison
27	Case-base
28	Case study
29	Observational study
30	Or 23–29
31	8 and 16 and 22 and 30

### 2.4 Selection of studies

Predefined standard eligibility criteria will be built before the study selection. Two independent reviewers will screen the titles and abstracts. All irrelevant studies or duplicates will be removed. Then, both 2 reviewers will examine the full texts of the remaining papers. Potential discrepancies on the eligibility criteria will be discussed with the help of another reviewer, and consistent decision will be made. We will show the results of study selection in a flowchart.

### 2.5 Data extraction and management

All information will be extracted from each eligible study by 2 independent reviewers according to the predefined data extraction form. The extracted information comprises of title, first author, year of publication, region, inclusion and exclusion criteria, sample size, diagnostic criteria, patient characteristics, study setting, research design and methods, treatment and control details, outcome measurements, and funding information. Any discrepancies regarding the data extraction will be solved with the help of a third reviewer. If some missing data or insufficient information occurs during the period of data extraction, we will contact the original corresponding authors via emails to inquire that essential information.

### 2.6 Study quality assessment

Methodological quality of included studies will be assessed by 2 reviewers using Newcastle-Ottawa Scale. If there are differences between 2 authors, we will invite a third author to solve through discussion, and a final decision will be reached.

### 2.7 Measures of treatment effect

We will use mean difference and 95% confidence intervals to express continuous data, and will utilize risk ratio and 95% confidence intervals to calculate dichotomous data.

### 2.8 Heterogeneity assessment

Heterogeneity analysis will be performed using *I*^2^ statistic. The *I*^2^ values represent a measure of variation in percentage across included studies. A value of *I*^2^ *≤* 50% suggests homogeneity, whereas value of *I*^2^ *>* 50% represents large heterogeneity, respectively.

### 2.9 Statistical analysis

We will use RevMan 5.3 software to carry out statistical analysis. A fixed-effect model will be exerted if the heterogeneity is homogeneity, and we will also plan to perform meta-analysis if sufficient eligible studies are included. A random-effect model will be applied if large heterogeneity is identified. Then, we will conduct subgroup analysis to check possible reasons for such high heterogeneity. If we can still identify obvious heterogeneity after subgroup analysis, we will report narrative summary instead of meta-analysis.

### 2.10 Additional analysis

#### 2.10.1 Subgroup analysis

We will conduct subgroup analysis based on the different characteristics, interventions and controls, and outcomes.

#### 2.10.2 Sensitivity analysis

We will perform sensitivity analysis to check the robustness of outcomes by removing low quality studies.

#### 2.10.3 Reporting bias

When there are sufficient studies (normally at least 10 studies), funnel plot and Eggers regression test will be conducted to identify reporting bias.^[21,22]^

### 2.11 Ethics and dissemination

This study does not need ethical approval, because we will not analyze individual patient data. This study is expected to be published at a peer-reviewed journal.

## 3 Discussion

Previous studies have reported that SPI is effective on PD in CMS with ELD. However, their findings are still inconsistent. In addition, no systematic review has explored the effect of SPI on PD in CMS with ELD. Thus, this study firstly investigates the effect of SPI on PD in CMS with ELD systematically and comprehensively. We search both electronic databases and other literature sources to avoid missing potential studies. Then, 2 reviewers independently carry out study selection, data collection and study quality assessment. We will invite a third experienced reviewer to solve any confusion between 2 reviewers through discussion. A final decision will be made after discussion. The results of this study may provide helpful evidence of SPI on PD in CMS with ELD.

## Author contributions

**Conceptualization:** Hong-li Wen, Chen Feng, Xiao-wei Li.

**Data curation:** Hong-li Wen, Xiao-wei Li.

**Formal analysis:** Shu-ling Zhang, Xiao-wei Li.

**Investigation:** Hong-li Wen, Xiao-wei Li.

**Methodology:** Chen Feng, Shu-ling Zhang.

**Project administration:** Xiao-wei Li.

**Resources:** Hong-li Wen, Chen Feng, Shu-ling Zhang.

**Software:** Hong-li Wen, Chen Feng, Shu-ling Zhang.

**Supervision:** Xiao-wei Li.

**Validation:** Hong-li Wen, Chen Feng, Shu-ling Zhang, Xiao-wei Li.

**Visualization:** Hong-li Wen, Shu-ling Zhang, Xiao-wei Li.

**Writing – original draft:** Hong-li Wen, Chen Feng, Shu-ling Zhang, Xiao-wei Li.

**Writing – review & editing:** Hong-li Wen, Chen Feng, Xiao-wei Li.
